# Digital documentation of individual treatment data and its relation to veterinary slaughter lesions in pigs

**DOI:** 10.5194/aab-68-589-2025

**Published:** 2025-09-24

**Authors:** Hannah Görge, Imme Dittrich, Thore Wilder, Nicole Kemper, Joachim Krieter

**Affiliations:** 1 Institute of Animal Breeding and Husbandry, Christian-Albrechts University, Olshausenstraße 40, 24098 Kiel, Germany; 2 Chamber of Agriculture of Schleswig-Holstein, Gutshof 1, 24327 Blekendorf, Germany; 3 Institute of Animal Hygiene, Animal Welfare and Farm Animal Behavior, University of Veterinary Medicine Hannover, Foundation, Bischofsholer Damm 15, 30173 Hanover, Germany

## Abstract

Legislation in the European Union demands the documentation of on-farm treatments and the documentation of lesions found at slaughter. The fulfilment of these documentation duties in a digitalised form allows further interpretation of the data. The aim of this study was to use a digital system for the documentation of on-farm treatments to determine the status quo of a farm's health status. Furthermore, relations between individual treatments and lesions found at slaughter were investigated. The data were obtained between August 2020 and September 2022 from a combined pig farm in northern Germany using a digital tool to document treatment data and from the slaughterhouses to which the pigs were delivered. The majority of individual treatments were observed in the fattening unit with 42.4 % of all individual treatments, followed by the rearing unit (40.4 %) and the piglet production (17.2 %). The most frequent reason for individual treatments was locomotor system diseases in all units (45.5 to 47.4 %), which occurred throughout the whole time of data collection. Gastrointestinal diseases represented the second most frequent individual treatment reason in the piglet production. Runt pigs in the rearing unit appeared in peaks, while tail lesions in the fattening unit seemed to occur especially in the colder months of the year. 18.2 % individuals received individual treatments. At the slaughterhouse, lesions were recorded for 18.1 % of the individuals. Lesions of the respiratory tract were found in 15.5 % of the individuals. The relation between slaughter lesions and individual treatment data was investigated using logistic regression models. In comparison to individual pigs which did not receive individual treatments, for individuals which received individual treatments in the early stage of life an odds ratio (OR) = 1.5 (95 % confidence interval (95 % CI): 1.2 to 2) and for individuals which received individual treatments in the later stage of life an OR = 1.9 (95 % CI: 1.4 to 2.5) were obtained. To conclude, there were higher risks for individuals which received individual treatments in the early or later stage of life but not for those who received treatments in both stages of life. Digitalised data of treatments can bring value to pig farming in regards to the overview they can provide.

## 1 Introduction

The emerging risk of antimicrobial resistance and increasing food safety regulations have forced several documentation duties on the farmer and the industry. As part of these duties, the documentation of treatments following Regulation (EU) 2019/06 on the use of veterinary medicinal products and the documentation of lesions found at slaughter following Regulation (EC) 854/2004 were set in place.

In the era of digitalisation, in which precision livestock farming and the Internet of things have emerged and shifted ever more into the focus of farm managing practices, the potential of these documents to provide additional value is yet to be discovered (Morrone et al., 2022). Digitalised data can be used to interpret the past, to identify matters in the present and to predict the future (Eastwood et al., 2019; Janssen et al., 2017; Wolfert et al., 2017). For example, the main diseases that require treatments can be pointed out for different production areas, and seasonal appearances of disease occurrences can be reduced with the implementation of preventive measures.

Studies have emerged investigating the potential of documented slaughter lesions as part of health monitoring schemes and disease surveillance (Correia-Gomes et al., 2017; Knage-Rasmussen et al., 2015; Nielsen et al., 2017). The potential lies especially in the cost-effective generation of data when compared to on-farm welfare assessments, since the data have to be documented by the law regardless. Nevertheless, Nielsen et al. (2015) pointed out that using slaughter inspection data for the cause of health monitoring should be used with caution since they were not originally intended for this purpose. To reflect on a herd's welfare status, on-farm data should be considered, additionally, especially since animal welfare is defined not only by physical integrity at one specific moment but rather by the balance of positive and negative experiences over a period of time (Carenzi and Verga, 2009). The Welfare Quality animal welfare assessment protocols are based on the four welfare principles “good feeding”, “good housing”, “good health” and “appropriate behaviour” (Dalmau et al., 2009). For “good health”, several studies have integrated on-farm treatment and antimicrobial use data into assessments, mainly using veterinary records (Bonde et al., 2005; Knage-Rasmussen et al., 2015). Nevertheless, the need to integrate on-farm data recording with veterinary recording has been highlighted, especially since there could be a discrepancy between prescribed and applied medication (Bracken, 2017; Menéndez González et al., 2010; Merle et al., 2012; Trauffler et al. 2014). Furthermore, Merle et al. (2012) observed digital data to be more accurate than handwritten documentation.

Therefore, the aim of this study was to use a digital system to document treatments linked to the individual pig at the time of application. Thus, the information of treatments presented in a digital format was used to determine the status quo of the investigated farm's health status, e.g. through the determination of treatment frequencies and treatment occurrences throughout the year. Additionally, treatment information was put into relation with veterinary inspection data from the slaughterhouses linked to an individual animal. The animal's life from birth to slaughter could be investigated by placing special emphasis on treatments.

## 2 Material and methods

### 2.1 Animals and housing

Data were collected at a German pig farm located in Schleswig-Holstein. The pig farm is a combined breeding and finishing farm, operating as a closed system and replacing sows with self-produced gilts. All pigs were housed according to the EU Council Directive 2008/120/EC, EU Council Directive 2010/63/EC and the “German Order for the Protection of Production Animals used for Farming Purposes and other Animals kept for the Production of Animal Products” (TierSchNutztV; Bundesministerium der Justiz und für Verbraucherschutz, 2021). Varying numbers of productive crossbred breeding sows (Large White × Landrace) with a mean of 147 sows were counted each day. They were artificially inseminated, testing offspring for the insemination organisation (GFS, Ascheberg, Germany), with the breeds of Duroc and Piétrain, as well as Large White and Landrace, for breeding and replacement purposes. Farrowing took place in farrowing compartments with plastic flooring. In four of the five farrowing compartments, the sows were fixated in farrowing crates. These compartments contained 10 to 12 pens each with slatted flooring. In the fifth farrowing compartment containing three pens, the crates were opened 5 d after farrowing. The floor was mostly slatted, except for a small area along the wall and the separated piglet nest, in which the floor was solid. The farm ran a 3-week farrowing cycle with a suckling period of 28 d. To test the offspring, every piglet received a radio frequency identification (RFID) ear tag on the first day of life, which was removed after identification at slaughter. The piglets' tails were not docked, and castration of the male piglets was carried out under injection anaesthesia during the first week of the piglets' lives. During the suckling period, the piglets were additionally fed with dry feed. The temperature in the farrowing departments was kept at 21 °C at farrowing and regulated down to 20 °C during the suckling period. After weaning, the piglets stayed in the same unit for 3 more days and were then moved to the rearing unit. The rearing unit was locally separated from the piglet production, and the pens were equipped with part slatted and part solid plastic flooring. Here, the piglets were kept in groups of 40 to 45 individuals. During the first 14 d of the rearing period, the piglets stayed in a compartment with a piglet nest, where the temperature was kept at 33 °C. In this compartment, the overall temperature was automatically regulated from 24.5 to 21.5 °C over the course of the 14 d. Afterwards, the piglets were transferred to compartments without piglet nests in which the temperature was kept at 22.5 °C. In both compartments, the piglets were fed with moist feeding ad libitum with a 3:1 animal to feeding place ratio. At an age of 63 to 67 d, the piglets were transferred into the fattening unit, which was locally separated again and had slatted concrete flooring. In the fattening unit the temperature was automatically regulated from 25 °C to 18 °C during the period of fattening. The unit contained 10 compartments, out of which 8 had 12 pens each and 2 contained 4 pens each. Each pen had a size of 2.5 m by 5 m. 15 fattening pigs were kept in each pen (stocking density: 0.83 m^2^ per pig). Since April 2022, only 13 fattening pigs have been kept in each of the pens (stocking density: 0.96 m^2^ per pig). The fattening pigs were restrictively fed with liquid feed with a 1:1 animal feeding place ratio three times a day at 05:00, 10:30 and 14:30 CET (central European time). The pigs stayed for 3–4 months at this location until slaughter (mean slaughter age: 178 d).

### 2.2 Treatment routine

Vaccinations and anthelmintic treatments were carried out routinely as prophylactic measures. The piglets were vaccinated against *Lawsonia intracellularis*, *Mycoplasma hyopneumoniae* and *Porcine circovirus*. Gilts were vaccinated against *Actinobacillus pleuropneumoniae, Erysipelas, Influenza, Mycoplasma hyopneumoniae, Parvovirus* and *Porcine circovirus*. Gestating sows were vaccinated against *Clostridia, Escherichia coli, Erysipelas* and *Parvovirus*. The anthelmintic treatments were carried out for the sows when brought into the farrowing pen and for the fattening pigs when brought into the fattening unit. In all units, daily animal observations were carried out, and pigs showing signs of illness, e.g. lameness, tail lesions or coughs, were treated as advised by the supervising veterinarian.

### 2.3 Data collection

For the data collection, a digital system was used from August 2020 to September 2022 to document the treatments at the time of application. The V-ETIC system (Henke Sass Wolf GmbH, Tuttlingen, Germany) operates with a cloud-based system which connects to a mobile application. The mobile application is connected via Bluetooth to an RFID reader, which is attached to a self-filling syringe. At the time of administration of a treatment, the RFID reader identifies the animal's RFID ear tag, and the treatment data linked to the individual are saved on the mobile device. The mobile application in combination with the RFID reader can be used offline in the stable but needs a WiFi connection to synchronise with the cloud-based system. The treatments are provided as treatment lists. A mobile phone and a RFID reader were placed at each of the three production areas piglet production, rearing and fattening unit to perform the digital treatment documentation, without risking disease transmission from one unit to another. The treatment routine was not changed due to the system implementation. Between August 2020 and January 2022, the farm was visited once a week to check for completeness of documentation and give support in system use. The digital documentation data were compared to the simultaneous handwritten documentation and completed when, for example, treatments were missing. Group treatments were not feasible to be documented with a direct link to the individual animal. After finishing the farm visits in January 2022, the treatment data were extracted from the cloud-based system.

Lists were created on the first day of the piglets' lives by the farm staff using another digital device (WORKABOUT PRO™ 3, Psion Teklogix Inc., Mississauga, Canada), into which the link of the piglet's RFID, birthdate and weight was deposited.

In addition to the on-farm treatment data, the insemination organisation provided the veterinary slaughter inspection data from all three slaughterhouses to which the pigs were delivered. The veterinary slaughter inspection in Germany is standardised following the General Administrative Regulation for Food Hygiene (AVV LmH, Die Bundesregierung, 2009) and documented as required by Regulation (EC) No. 854/2004.

**Figure 1:**
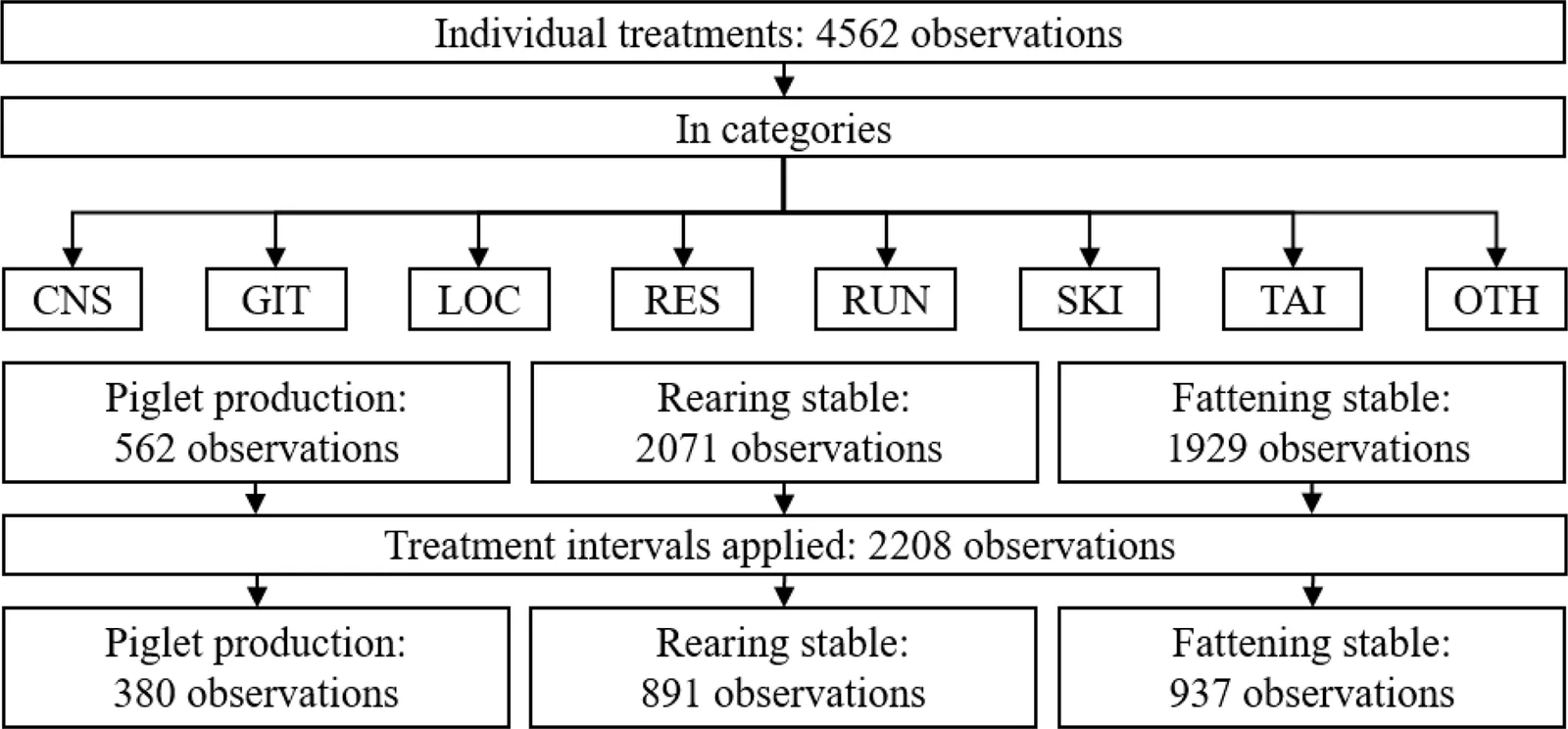
Display of observations used for analyses in the present study. The treatments were assigned to eight categories including treatments of the central nervous system (CNS), the gastrointestinal tract (GIT), the locomotor system (LOC), the respiratory system (RES), runt pigs (RUN), skin lesions (SKI), tail lesions (TAI) and other treatments (OTH). Treatment intervals allowed the separation of ongoing treatments from new treatments with 14 d for LOC and 6 d for the other categories.

### 2.4 Data restriction and descriptive analyses

All analyses were carried out using the statistical software SAS^®^ 9.4 (SAS Institute Inc., 2018).

In August 2020, the beginning of data collection, only treatments in the area of piglet production were documented due to system malfunctions that were fixed afterwards. The treatment documentation was then extended to the other production areas in September. In December 2020, the system at the rearing unit went through technical issues, during which some data were lost. Due to further technical issues, data collection stood still in April and May 2021, and data documented between 27 May and 2 June 2022 in the piglet production and rearing unit were lost.

Overall, there were 5551 observations of treatments in the production areas piglet production, rearing unit and fattening unit (Fig. 1). To trace animals from birth to slaughter, only individual treatments linked to individual animals were considered, resulting in 4562 observed individual treatments. In the rearing unit, group treatments were carried out for 51 groups including 2069 individuals from October to November 2020, April to July 2021 and November 2021. In the fattening unit, group treatments were carried out for 5 compartments including 720 individuals in April and November 2021. The group treatments targeted respiratory diseases exclusively in the fattening unit and predominantly in the rearing unit. All group treatments were excluded from the analyses due to missing linkage to individual animals. No group treatments were carried out in piglet production. The individual treatments were assigned to eight treatment categories depending on the underlying treatment indication. The categories included treatments of the central nervous system (CNS), the gastrointestinal tract (GIT), the locomotor system (LOC), the respiratory system (RES), runt pigs (RUN), skin lesions (SKI), tail lesions (TAI) and other treatments (OTH) (Table 1).

**Figure 2:**
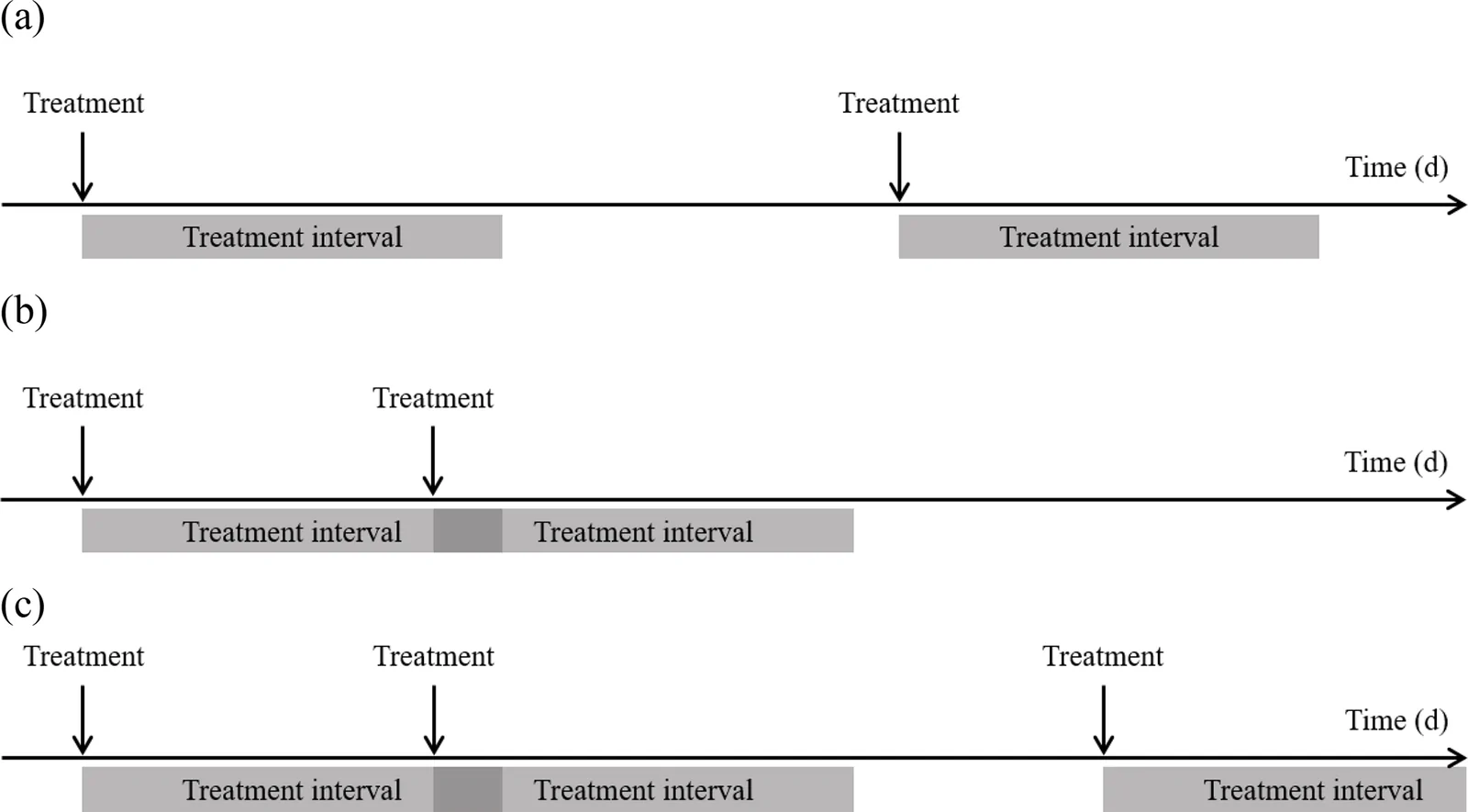
Treatment intervals determine whether additional treatments in the same treatment category count as ongoing treatments or as new treatments: **(a)** displays two separate treatments; **(b)** displays one treatment with an ongoing treatment. The treatment interval is prolonged, and **(c)** again displays two separate treatments, where the first treatment includes an ongoing treatment and the treatment interval is prolonged.

Treatment intervals were defined to determine whether a following treatment of the same individual was a treatment of a new disease or whether it was counted as an ongoing treatment of the previous disease (Fig. 2). For this purpose, distribution analyses of the difference in days between treatments were conducted. Taking treatment intervals applied in other literature and the duration animals might be affected by the different disease categories into account, a time period within the 90th percentile was selected (Bareille et al., 2003; Baumgärtner and Gruber, 2020; Buttchereit et al., 2012; Christensen et al., 1994; Dunlop et al., 1998; Houe et al., 2011; Smith, 1988). After applying the treatment intervals, 2208 observations of treatments were left, forming Dataset 1.

**Table 1:** Treatment indications placed into categories: of the central nervous system (CNS), the gastrointestinal tract (GIT), the locomotor system (LOC), the respiratory system (RES), runt pigs (RUN), skin lesions (SKI), tail lesions (TAI) and other treatments (OTH).

Category	Indication
CNS	Head tilt
	Muscle twitching
	Paddling
GIT	Diarrhoea
	Intestinal obstruction
	Rectal prolapse
LOC	Claw injury lameness
	Swollen claw
	Swollen joint
	Swollen joints
	Swollen leg
RES	Cough
	Dyspnoea
	Pneumonia
	Pumping
RUN	Thin
	Weak
SKI	Abscess
	Eczema
	Red spots
	Wound
TAI	Tail-biting lesion
	Tail tip necrosis
OTH	Apathy
	Aural haematoma
	Blue ears
	Fever
	Abdominal hernia
	Inappetence
	Jaw fracture
	Red eye
	Umbilical infection

With the consideration of the lists generated on the first day of the piglets' lives, Dataset 2 was formed. Due to technical malfunctions, the lists generated on the first day of the piglets' lives were not complete, resulting in an exclusion of 10.8 % of the individuals who received treatments in Dataset 2.

Dataset 3 included the additional information on the veterinary slaughter inspection data. One of the three slaughterhouses was excluded from the analyses due to the small number of observations ( n=133 ), resulting in the integration of slaughterhouse A ( n=3310 ) and B ( n=320 ) into the analyses. At slaughter, the RFID ear tags of 33.2 % of all slaughtered pigs were not read, because the readers were neglected to be switched on during slaughter routine or because ear tags were lost on the farm or during transportation. Additionally, data exclusion had to take place on five slaughter dates between July and October 2022 on which pigs were delivered alongside pigs without any information about treatments, which could not be differentiated. Therefore, individuals which received treatments could not be integrated in the dataset used for the statistical analyses (Dataset 3).

For the statistical analyses, pigs that had been born from August 2020 were included. Hence, the first slaughter date to be used for analyses was 27 January 2021. The last slaughter date included was 12 October 2022, since the treatment data collection stopped at the end of September 2022, resulting in 3630 included observations. The slaughterhouse lesions were then aggregated into five categories: respiratory health (lesions of lung, pleura and pericardium), other organ health (lesions of liver, intestines and peritoneum), health of the extremities (arthritis, bursitis), integrity of the carcass (lesions of the skin, including tail lesions and necroses) and general health (including all lesions). An overview of the datasets and the analyses they were used for are displayed in Table 2.

**Table 2:** Numbers and nature of datasets used for the different analyses. OR = odds ratios, LSM = least square means, SE = standard errors.

Dataset	Numbers of observations	Nature of dataset	Used for
Dataset 1	2208	Treatment data	Descriptive analyses of treatment categories per production area, Frequencies of the treatment categories, number of repeated treatments, distribution of treatments throughout the data collection period
Dataset 2	9890 = Piglet production 8976 = Rearing unit 8144 = Fattening unit8144 = Overall	Treatment data and lists generated on the first day of the piglets' lives	Descriptive analyses of treatment frequency of individuals for the treatment categories and production areas
Dataset 3	3630	Treatment data, lists generated on the first day of the piglets' lives and veterinary slaughter inspection data	Statistical analyses with logistic regression models: significance, OR, LSM, their SE and their differences

### 2.5 Statistical analyses

For the statistical analyses of the relation between veterinary slaughter inspection data and age at treatment, Dataset 3 was used. The veterinary slaughter lesions were presented as binary data defining the absence or presence of lesions. Lesions of the pleura and lung were classified in the lists provided as negative, marginal, moderate and severe. The scorings of the lesions were aggregated to a binary score: (0) absent (negative, marginal) and (1) present (moderate, severe). The variable “treatment and stage of life” (TS) was generated: (00) no treatment, (10) treatment in the early stage of life (piglet production and rearing unit), (01) treatment in the later stage of life (fattening unit) and (11) treatment in both stages of life. To integrate potential effects of birth group and season, the variable birth group was created. One birth group included three subsequent farrowing groups.

Logistic regression models following logit functions were used to carry out the analyses. The dependent variables, in this case the veterinary slaughter lesions in the categories general health, respiratory health, other organ health, health of the extremities or integrity of the carcass, were explained by the three fixed effects TS, slaughterhouse (A, B) and birth group (A to J). The fixed effects were included in the model in a stepwise manner, considering Akaike's information criterion (AIC) and the deviance divided by its degrees of freedom (target value = 1). For the interpretation of the data, 5 % were used as level of significance, the odds ratios (ORs), the least square means (LSMs), and their standard errors (SEs), and their differences were considered, with the level of significance adjusted by Bonferroni.

## 3 Results

### 3.1 Descriptive analyses

#### 3.1.1 Treatment intervals and frequencies

For the LOC category, the treatment interval of 14 d was defined, as 90 % of the treatments were separated by less than 14 d. In the other categories (CNS, GIT, RES; RUN, SKI, TAI and OTH), a uniform solution was found with an interval of 6 d derived from rearing and fattening since the majority of treatments (82.1 %) were carried out in these production areas.

Overall, 18.2 % of all pigs going through all production areas were treated individually (Table 3). The LOC category occupied the most observations, representing 46.9 % of the treatments over all units. The next most frequent treatment reasons were in the RUN category (12.6 %), followed by the TAI (12.5 %) and RES (10.7 %) categories.

**Table 3:** Treated individuals in the production areas piglet production, rearing unit and fattening unit. Displayed are categories with > 1 % treated pigs. The categories include all treatments (ALL) and treatments of the central nervous system (CNS), the gastrointestinal tract (GIT), the locomotor system (LOC), the respiratory system (RES), runt pigs (RUN) and tail lesions (TAI).

Production area	Category	Treated pigs	Proportion (%)
Overall ( n=8144 )	ALL	1480	18.2
Piglet production ( n=9890 )	ALL	358	3.62
	GIT	134	1.35
	LOC	161	1.63
Rearing unit ( n=8976 )	ALL	705	7.85
	LOC	351	3.91
	RUN	172	1.92
Fattening unit ( n=8144 )	ALL	639	7.85
	LOC	331	4.06
	RES	122	1.50
	TAI	164	2.01

Out of all the treatments, 17.2 % were applied in the piglet production. In the rearing unit 40.4 % of all treatments were administered, and the most treatments (42.4 %) were administered in the fattening unit. In the piglet production 3.6 % and in both the rearing and fattening unit 7.85 % of all pigs received treatments.

**Figure 3:**
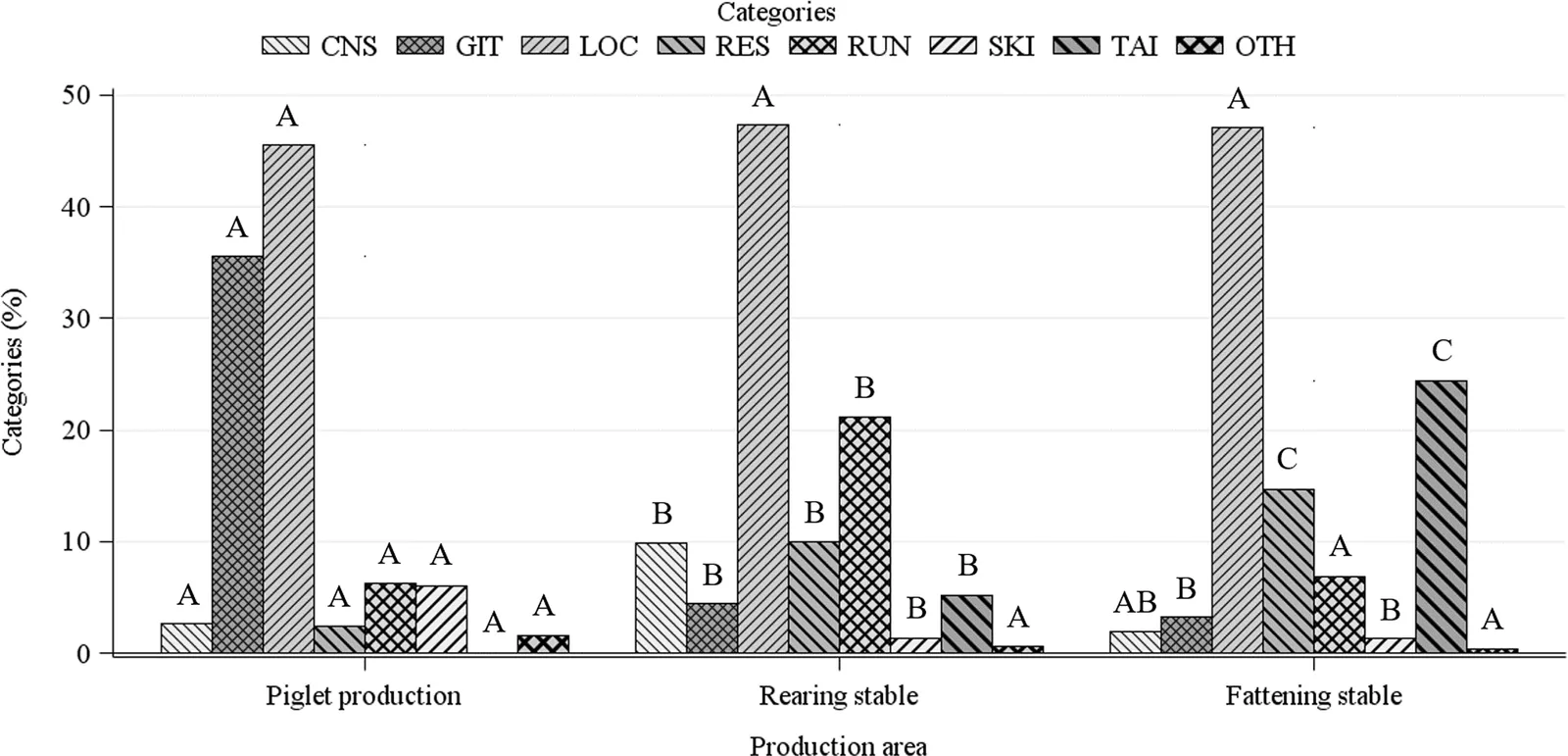
Proportion of treatment categories in the production stages piglet production, rearing unit and fattening unit. The categories include treatments of the central nervous system (CNS), the gastrointestinal tract (GIT), the locomotor system (LOC), the respiratory system (RES), runt pigs (RUN), skin lesions (SKI), tail lesions (TAI) and other treatments (OTH). A, B and C indicate significant differences between production stages within treatment categories.

The proportions of treatment categories for each production area are displayed in Fig. 3. It highlights that, in addition to LOC treatments, which were present from 45.5 % to 47.4 %, the treatment focus was put on different treatment categories depending on the production area. While it was put on the GIT category (35.5 %) in the piglet production and on the RUN category (21.2 %) in the rearing unit, in the fattening unit the focus shifted towards the TAI category (24.4 %).

Regarding repeated treatments of individuals in the LOC category, 6.1 % and 5.8 % of the treated rearing piglets and the treated fattening pigs received more than one treatment, respectively. While the other categories over the production areas presented low numbers of repeated treatments, the TAI category in the fattening unit with 15.3 % of repeatedly treated individuals can be pointed out.

#### 3.1.2 Distribution of treatments over time

When looking at the treatments of all production areas, a mean value of 2.75 individual treatments was carried out over all the units each day. A number of individual treatments per day were carried out: in the piglet production 0.43, in the rearing unit 1.08 and in the fattening unit 1.23 (Table 4). Over all the units the most treatments were counted in January 2022 and the fewest treatments in August 2022.

**Table 4:** Treatments per day for the different categories in the production areas with 760 d with observations. Displayed are categories with mean treatments > 0.1 d^−1^. The categories include all treatments (ALL) and treatments of the central nervous system (CNS), the gastrointestinal tract (GIT), the locomotor system (LOC), the respiratory system (RES), runt pigs (RUN) and tail lesions (TAI).

Production area	Category	Mean	Minimum	Maximum	Standard deviation
Overall	ALL	2.75	0	32	4.06
Piglet production	ALL	0.43	0	27	1.90
	GIT	0.11	0	27	1.50
	LOC	0.23	0	14	0.96
Rearing unit	ALL	1.08	0	18	2.14
	CNS	0.12	0	7	0.54
	LOC	0.56	0	14	1.29
	RUN	0.25	0	13	1.08
Fattening unit	ALL	1.23	0	24	2.39
	LOC	0.58	0	9	1.11
	RES	0.18	0	24	1.30
	TAI	0.30	0	14	1.16

**Figure 4:**
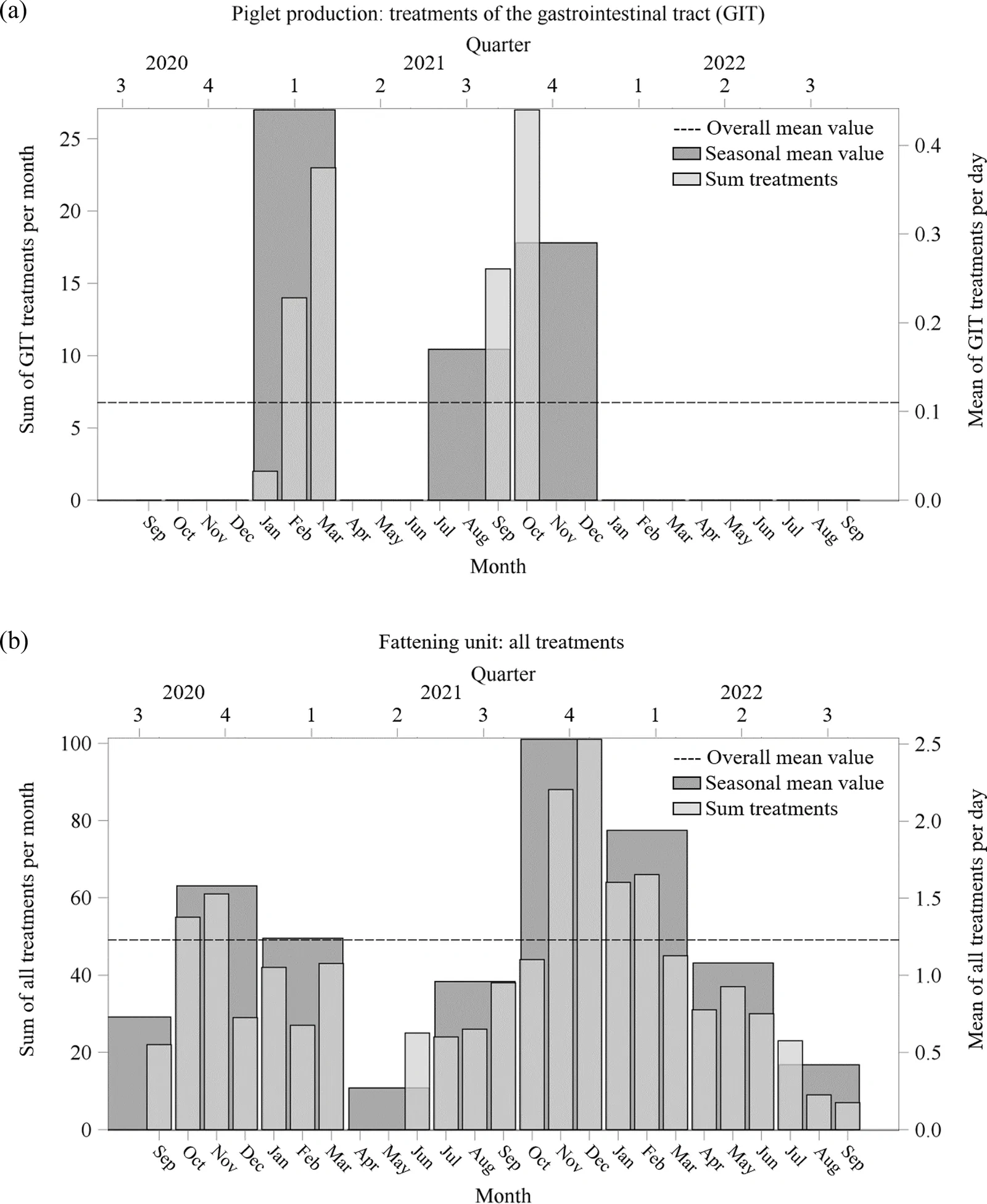
Distribution of individual treatments from September 2020 to September 2022 **(a)** for treatments of the gastrointestinal tract (GIT) in the piglet production and **(b)** for all treatments in the fattening unit.

Treatments within the GIT category in the piglet production appeared only in the periods between January and March 2021 and September and October 2021, when the most treatments were counted in this production area (Fig. 4a).

By contemplating all treatments in the rearing unit, accumulated treatments occurred in September to November 2020, June to August 2021 and around March 2022. Peaks within the RUN category occurred in November 2020, July and August 2021, and March and May 2022. Looking at the LOC category, there were peaks in treatments from June to August in 2021 and 2022 and another peak in January to March 2022. Therefore, treatments occurred throughout the whole period of data collection, and no definite trends were detected.

In the fattening unit, the highest treatment numbers occurred in November and December 2021. There were lower treatment numbers in the warmer months of the years compared to the colder months (Fig. 4b). Treatments in the LOC category peaked in both October 2020 and in December 2021, while treatments in the TAI category prominently peaked in December 2021.

#### 3.1.3 Lesions at slaughter

Lesions in the general health category were found in 18.1 % of all pigs. Over all pigs, 15.5 % showed lesions in the respiratory health category, 2.64 % in the other organ health category, 0.28 % in the health of the extremities category and 1.35 % in the integrity of the carcass category. Due to the low prevalence in the other categories, only the general health category and the respiratory health category are considered in the following statistical analyses.

The proportions of variable TS in Dataset 3 are presented as follows: of all pigs arriving at slaughter, 17.4 % were treated at least once in their lives, similar to the results gathered from Dataset 2, and 18.2 % were treated at least once in their lives (Table 3). Treatments in the early stage of life were administered to 8.76 %, treatments in the later stage of life were administered to 7.55 % and treatments in both stages were administered to 1.07 % of all pigs arriving at slaughter.

### 3.2 Logistic regression models

#### 3.2.1 General health model

For the logistic regression model regarding the general health at slaughter (GHM), the smallest AIC was achieved after integrating the variables TS, slaughterhouse and birth group in a stepwise manner. The deviance moved towards 1 (1.24 to 1.02). The Hosmer–Lemeshow goodness-of-fit test indicated good fit. The variables TS and birth group presented with p<0.001 and were considered to have a significant effect on the slaughter lesions.

**Figure 5:**
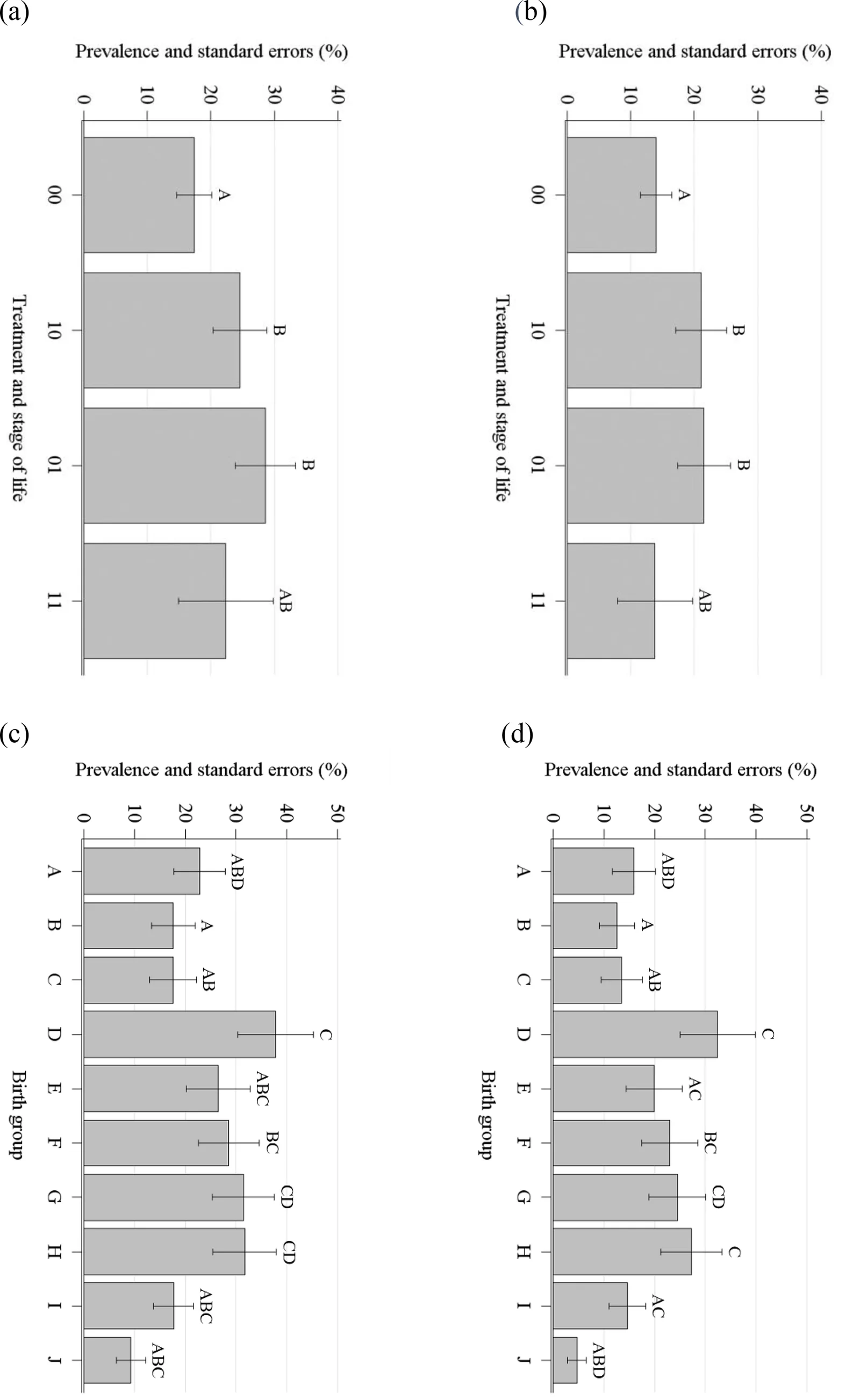
Least square means and standard errors for slaughter lesions of the variable “treatment and stage of life” **(a)** in the general health model (GHM) and **(b)** in the respiratory health model (RHM) and of the variable “birth group” **(c)** in the GHM and **(d)** in the RHM.

The LSMs and their differences are displayed in Fig. 5. In Fig. 5a, it is visible that having received a treatment in the early ( p=0.016 ) or the later ( p<0.001 ) stage of life is statistically different to having received no treatment. The LSMs were determined as 0.25 (SE: 0.04) for pigs treated in the early stage of life, 0.29 (SE: 0.05) for pigs treated in the later stage of life, 0.22 (SE: 0.07) for pigs treated in both stages and 0.17 (SE: 0.03) for pigs that were not treated.

**Figure 6:**
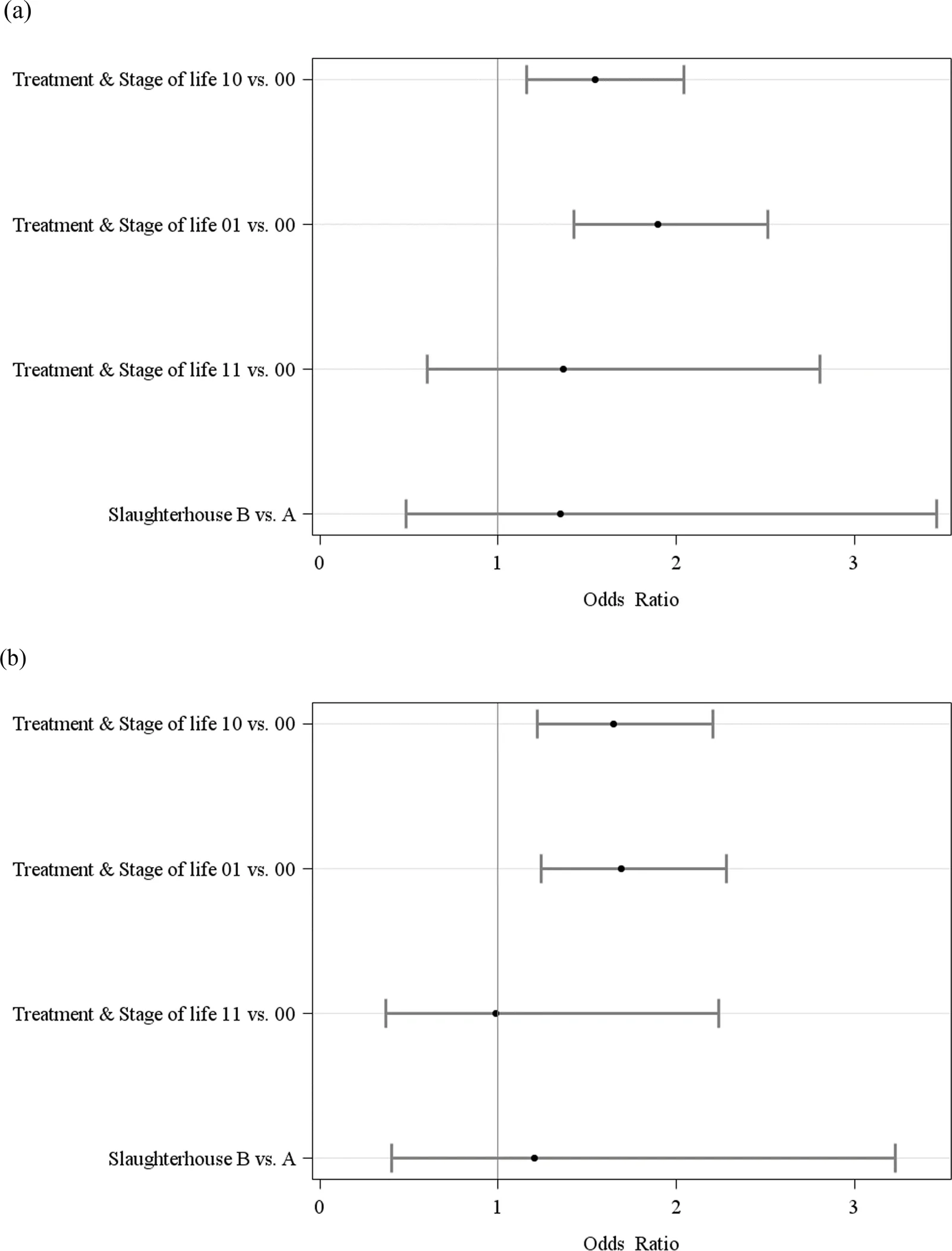
Odds ratios for the variable treatment and stage of life and slaughterhouse **(a)** in the general health model and **(b)** in the respiratory health model.

The ORs, displayed in Fig. 6a, indicate higher risks of slaughter lesions of individuals having received treatments in the early (OR = 1.6, 95 % confidence interval (95 % CI): 1.2 to 2) and later (OR = 1.9, 95 % CI: 1.4 to 2.5) stages of life when compared to no treatments. This is not the case for individuals having received treatments in both defined stages of life.

Regarding the slaughterhouses, the ORs do not indicate higher or lower risks of lesions if the pigs were inspected at either slaughterhouse A or B. No statistical differences in the LSMs were found.

The LSMs for the birth groups ranged from 0.09 to 0.38, with the lowest value determined for birth group J (SE: 0.05) and the highest value for birth group D (SE: 0.04). Birth groups D, G and H, which present with the highest LSMs, differ significantly to birth groups B and C. The complexity of the differences in LSM of the birth groups is displayed in Fig. 5c.

The ORs of the birth groups are determined in comparison to the risk of birth group A. The ORs show that birth groups D (OR = 2.1, 95 % CI: 1.4 to 3.1), G (OR = 1.6, 95 % CI: 1.1 to 2.1) and H (OR = 1.6, 95 % CI: 1.2 to 2.2) have higher risks, whereas the other birth groups present with similar risks of slaughter lesions.

#### 3.2.2 Respiratory health model

In the respiratory health model (RHM), the best-fitting AIC could be obtained after integrating the variables TS, slaughterhouse and birth group in a stepwise manner, as well as a deviance close to 1. The Hosmer–Lemeshow goodness-of-fit test also indicated good fit. Overall the RHM is similar to the GHM; the differences are pointed out in the following.

In Fig. 5b, it is visible that having received a treatment in the early ( p=0.005 ) or the later ( p=0.004 ) stage of life is statistically different to having received no treatment. The LSMs presented with 0.21 (SE: 0.04) for pigs treated in the early stage of life, 0.21 (SE: 0.04) for pigs treated in the later stage of life, 0.14 (SE: 0.06) for pigs treated in both stages and 0.14 (SE: 0.03) for pigs that were not treated.

Both risks of lesions at slaughter were present if treatments in either one of the life stages were performed with an OR = 1.7 (95 % CI: 1.2 to 2.2 for early treatments; 95 % CI: 1.3 to 2.3 for late treatments) (Fig. 6b).

The LSM for the birth groups ranged from 0.05 (birth group J, SE: 0.02) to 0.32 (birth group D, SE: 0.07). Birth groups D and H, which present with the highest LSM, differ significantly to birth groups A, B, C and J. The complexity of differences in LSM is displayed in Fig. 5d.

In this model, birth group F also presents with a higher risk (OR = 1.6, 95 % CI: 1.1 to 2.3) of slaughter lesions. It is noticeable that especially for birth group D (OR = 2.5, 95 % CI: 1.6 to 3.9) a higher OR is present than in the general health model. Additionally, birth group J has a lower risk (OR = 0.3, 95 % CI: 0.1 to 0.9) for slaughter lesions.

## 4 Discussion

### 4.1 Treatment data

This study focusses on the relation of treatment data and slaughter lesions, to investigate the hypothesis that pigs which never required treatments were healthier at slaughter than pigs which were treated at least once.

Whether treatment data can fully reflect the disease occurrence of a herd is controversially discussed. Kelton et al. (1998) suggested it can lead to over-reporting when diseases require more than one treatment or when treatments are utilised for prophylactic measures. To prevent over-reporting in the context of ongoing treatments in the present study, treatment intervals were used, following the approaches of other studies (Christensen et al., 1994; Dunlop et al., 1998; Houe et al., 2011). With regard to over-reporting due to prophylactic measures, vaccinations and anthelmintic treatments were excluded from the analyses. Other prophylactic treatments did not take place during the time of data collection. In this context, following the guidelines for the prudent use of veterinary antimicrobial drugs, the prophylactic use of antimicrobials must be avoided in Germany (Federal Ministry of Food and Agriculture, 2010), and since January 2022, the prophylactic and metaphylactic use of antibiotic treatment has been prohibited in the European Union and reserved for justified exceptions, e.g. when risks of infections are very high, major consequences are expected and no alternatives are available, following Regulation (EU) 2019/06. In contrast to over-reporting, under-reporting might take place if individuals are considered healthy, even though they show symptoms of disease, or the disease is simply not treated (Kelton et al., 1998; Weber et al., 2015).

In the context of animal welfare, it is known that disease or the absence of disease reflects the welfare status and stress level of the animals (Sainsbury, 1986; von Borell, 2000). In the literature it is described that several data sources such as clinical observations combined with treatments and slaughter lesions should be considered to fully be able to reflect on a herd's welfare status (Bonde et al., 2005; Wadepohl et al., 2020). A full audit on clinical observations from independent observers as carried out in other studies is time-consuming, is expensive and only reflects on the health status of a herd at a single moment, where some clinical signs can easily be overlooked or underestimated by the observer because they are not shown constantly, e.g. coughing and diarrhoea (Nielsen et al., 2022; Petersen et al., 2008). For the assessment of lameness, every pig that does not move at the time of audit has to be motivated to move as practised in the study of Willgert et al. (2014).

Since it is mandatory to document treatments in the European countries, this study explored the possibilities of the mandatory documentation of on-farm treatment data in a digitalised form. To date, there are few studies relying on on-farm treatment data collection, as it is considered inaccurate and data are rarely available in a standardised format for comparative purposes. However, on-farm data collection has been described as being successful and feasible, especially when electronic documentation is utilised (Menéndez González et al., 2010; Merle et al., 2012; Trauffler et al., 2014). Because veterinary dispensary records are mostly thoroughly recorded, they are used more often to investigate the health status of herds (Bonde et al., 2005; Knage-Rasmussen et al., 2015). Nevertheless, they could potentially lead to an over-reporting of treatments, since there could be a discrepancy present between prescribed and administered medicines (Bracken, 2017).

In this study, malfunctions of the systems used on-farm and in the slaughterhouses led to loss of data. On-farm, technical malfunctions, which could not be fixed immediately, caused loss of treatment data in four time periods of between 1 week and 2 months of length. This highlights the need for technical improvements, before the system can be feasibly used on commercial farms. At the slaughterhouse, one-third of the RFID ear tags were not read; therefore, the data could not be linked to the individual animals. That is attributed to neglecting to switch on the readers during the slaughter routine or to the loss of ear tags on the farm and during transportation to the slaughterhouse. Caja et al. (2005) found 1.2 % of ear tags were lost during transportation to the slaughterhouse. Other literature found 4 % to 7 % of RFID ear tags to have been lost on the farm (Maselyne et al., 2014; Thölke and Wolf, 2022).

Additionally, the treatment number is too low because group treatments were not included in the data collection. Nevertheless, group treatments were administered mainly in the rearing unit and rarely in the fattening unit to treat respiratory diseases predominantly. Since European legislation forbids prophylactic and metaphylactic treatments, the pigs with the most severe disease symptoms were likely to have been treated individually before group treatments were initiated. This could be confirmed with the comparison of the distribution of treatments over time and the handwritten documentation of group treatments, as peaks of individual treatments within the categories occurred prior to the administration of group treatments. As only animals with severe disease symptoms were treated individually, animals with subclinical respiratory diseases may likely be only treated in group treatments but still show respiratory lesions at slaughter. This could explain the contrast between the on-farm individual treatment data and the respiratory lesion data of the slaughterhouse.

Furthermore, a limitation in terms of a “healthy worker effect” first described by McMichael (1976) is likely to be present in this study. Of course, individuals that died within the farrow-to-finish period could not be included in the analyses of the treatments and slaughter lesions; thus the investigated pigs may only represent the strong individuals of the farm.

### 4.2 Treatment frequencies

Within this study, 18.2 % of all pigs had undergone treatment once in their life. Nielsen et al. (2022) pointed out that there is a variation of treatment prevalence in conventional pig farms, finding prevalence of 5 to 13 % in their study, in which only data on the piglet production were integrated. As described by Hybschmann et al. (2011) for the example of gastrointestinal disorders, differences in treatment incidences are related not only to disease incidences but also to management factors, biosecurity measures, and treatment decisions in accordance with the individual farmers and veterinarians' attitude.

Raasch et al. (2018) found, in a sample size of 60 farrow-to-finish herds in Germany, the most treatments to be administered at the rearing unit, which would reflect the results of the present study if group treatments were considered. In the present study, the individual treatment numbers were slightly lower in the rearing unit than in the fattening unit. When contemplating the treated individuals per production area, 7.85 % of all pigs were treated in both units. Considering the periods, the pigs stayed 4–5 weeks in the rearing unit, while they stayed 3–4 months in the fattening unit; the same number of pigs had been treated in a shorter period in the rearing unit. Additionally, more treatments were applied at the fattening unit to the same number of individuals, which can be attributed to the relatively high numbers of repeated treatments within the TAI category in this production area.

### 4.3 Treatment focus of production areas

In the piglet production, the focus of treatments was on the LOC and GIT categories, which have also been the main indications for treatments in this production area in other studies (Christensen et al., 1994; Johansen et al., 2004; Nielsen et al., 2022; Raasch et al., 2018). Nielsen et al. (2022) found GIT treatments especially in piglets that were being cross-fostered and discussed it as being due to an increased potential for pathogen transmission, increased stress levels and disturbed feed intake. Since the high numbers in GIT treatments increase the overall treatment numbers for the piglet production when they occur, the role of infectious diseases is highlighted, especially for this production area. In this context, the importance of biosecurity measures, including cleaning and disinfection routines to avoid disease entrance and transmission, and their relation to treatments is underlined as it has been in other studies (Pandolfi et al., 2018; Postma et al., 2017; Raasch et al., 2018).

Focusing on the rearing unit, indications within the LOC and RUN categories played an important role. Raasch et al. (2018) reported mainly gastrointestinal and respiratory diseases in addition to lameness in this production area. They had a high proportion of orally administered treatments in their study, and Timmerman et al. (2006) described that oral treatments are mainly administered post weaning to treat gastrointestinal diseases, arthritis or respiratory diseases. Sarrazin et al. (2019) found that group treatments were mainly administered in the rearing unit in Europe. The present study did not take group treatments into account, and group treatments were administered to treat gastrointestinal and respiratory diseases, which explains the difference in results. Nevertheless, high numbers of treatments in the RUN category are present in this study. Explanations can be sought in the challenging process of weaning, during which piglets have to adjust to new surroundings, cope with unfamiliar piglets as they are regrouped and have to deal with nutritional challenges. This may lead to a low immune status of rearing piglets and an accumulation of diseases occurring at that time (Campbell et al., 2013; Weary et al., 2008).

In the fattening unit, the treatment focus in this study was on the LOC and TAI categories. Higher occurrences of tail biting in undocked pigs than in docked pigs, as well as in conventional farms when compared to wean-to-finish farming systems, have been found in other studies (Gentz et al., 2020; Schrøder-Petersen and Simonsen, 2001). Nevertheless, the consequences of tail docking in the form of acute and chronic pain should be highlighted and considered ethically (Valros and Heinonen, 2015). In the literature, a relation between decreasing temperatures and an increasing prevalence of tail lesions and lameness has been described (EFSA, 2007; Lee et al., 2020; Zoric et al., 2003), which concurs with the distribution of treatments over time in this study. A relationship between heat stress and tail lesions has been discussed as well (D'Eath et al., 2014; EFSA, 2007; Schrøder-Petersen and Simonsen, 2001). Since tail lesions may lead to intercurrent disease, for example respiratory disease, due to the spread of bacteria resulting in septicaemia or pyaemia, this could lead to the overall higher treatment numbers in this production area, when they occur (Schrøder-Petersen and Simonsen, 2001). The reduction of treatments towards the warmer months of the year 2022 may also have been related to the reduction of pigs per pen in April 2022. Meyns et al. (2011) described that less space per individual heightens the risk of disease occurrences. The relatively high numbers of repeatedly treated individuals in the TAI category might indicate that tail-biting management could be optimised by, for example, following tail-biting intervention protocols as proposed by Chou et al. (2019).

### 4.4 Lesions at slaughter

The reported slaughter lesions do not reflect the on-farm treatment reasons, since there were only small numbers of lesions other than respiratory-health-related lesions reported. This is in accordance with studies by Horst et al. (2020), Pandolfi et al. (2018) and Teixeira et al. (2016). Additionally, Bonde et al. (2010) found that parasitic, intestinal and heart disorders are likely to be underestimated at slaughter. Similar risk factors such as climate and housing factors may result in the presence of tail lesions, which were treated mainly during the fattening period, and respiratory lesions, which were reported frequently at slaughter (EFSA, 2007; Meyns et al., 2011; Stärk, 2000). Correia-Gomes et al. (2017) pointed out that it is difficult to assess tail damage due to the rapid speed of the lines and the position of the observer. Thus, respiratory lesions may be more likely to be reported.

### 4.5 Logistic regression models

Both statistical models describe a similar situation, since the GHM contains all the information of the RHM in addition to the slaughter lesions of the other categories, and the majority of slaughter lesions were documented in the respiratory health category.

The two slaughterhouses integrated in the models did not differ significantly, even though the number of observations differed. The exclusion of slaughterhouse B, to which relatively low numbers of pigs were sent, did not result in a difference in results of the models. The effects of different thresholds to document lesions by meat inspectors and the effects of slaughterhouses and farms have been discussed in other studies (Bonde et al., 2010; Eckhardt et al., 2009; Enøe et al., 2003; Hoischen-Taubner et al., 2011).

There is a positive relation present between treatments and lesions documented at slaughter, when treatments were performed either in the early or in the later stages of life but not when they were performed in both stages. The risk of lesions for individual pigs which received treatments in the later stage of life is higher in the GHM than in the RHM, which leads to the assumption that lesions which are not respiratory-health-related show more prominently when the diseases have recently occurred. Blocks et al. (1994) found slaughter lesions to be positively related to initiation of treatments especially when treatments were applied nearer to the slaughter date. Nevertheless, treatments in the early stages of life also posed higher risks of lesions at slaughter. The literature discusses the first 12 weeks in a piglet's life to be critical especially in developing gastrointestinal immunity. For a good and long-term maturation of the immune system, an immunosuppressive environment seems to be essential (Moeser et al., 2017). Early disease events might influence this development, resulting in higher susceptibility to disease in the pig's ongoing life.

Birth group J had the lowest LSM of slaughter lesions. When looking deeper into the treatment data of birth group J, it presents with the least number of treated pigs in the group. As mentioned before, this could be due to the reduction of pigs per pen in April 2022, since Meyns et al. (2011) described that space per individual correlated with disease occurrences. There are other differences between the birth groups, as shown in Fig. 5c and d. However, the effect birth group may comprise more effects like season, management or outbreaks of disease, which could not be included individually in the model. The individual influences of these effects could be investigated in more detail in future studies.

Treatments performed in both stages of life did not pose a statistically significant higher risk of slaughter lesions. There is a relatively low number of individual pigs which received treatments in both stages in Dataset 3. Salas-Eljatib et al. (2018) found that unbalanced datasets result in higher variabilities, which might result in the wide range of confidence limits of the OR and the high standard error of LSM, which is especially visible when contemplating the LSM in the GHM (Fig. 5a).

### 4.6 Limitations

While the findings provide valuable insights, limitations should be acknowledged. As mentioned, group treatments were not included in the analysis, because it was not possible to link the treatments to the individual animals. Since one reason for group treatments was respiratory diseases, these may be underestimated in this study. Additionally, data were lost on the farm, because malfunctions of the system could not be easily fixed. Moreover, identification of some animals at the slaughterhouse was not possible because of lost ear tags or because of neglecting the ear tag reader in the routine of the slaughterhouse. Therefore, some data could not be linked to the individual animal and had to be excluded from analysis. This was mitigated by prolonging the period of data collection to ensure a sufficient sample size. Furthermore, this study was conducted on a commercial farm. Thus, it was not possible to control all aspects of this study, as some measures were based on management decisions. As a consequence, there are effects which could not be included individually in the logistic regression models which may be part of the fixed effect birth group. As those effects cannot be distinguished in this analysis, the interpretation of the effect birth group is difficult. This could be further investigated in future studies conducted on research farms with controlled environments.

## 5 Conclusions

This study highlighted the value of digitalised treatment data, in special regard to the overview such data can provide. However, technical improvements are still needed to ensure reliability and to avoid data losses. The most frequent reasons for treatment of individual animals were in the category of the locomotor system and in the fattening unit within the category of tail lesions. By putting treatment data linked to the individual animal in relation to lesions reported at slaughter, a positive relation between treatments in the early or later stages of the individual's life was observed. However, a positive relation between treatments in both stages of the individual's life could not be confirmed.

## Data Availability

Data are available from the corresponding author upon reasonable request.
